# Therapeutic Efficacy of an ω-3-Fatty Acid-Containing 17-β Estradiol Nano-Delivery System against Experimental Atherosclerosis

**DOI:** 10.1371/journal.pone.0147337

**Published:** 2016-02-03

**Authors:** Dipti Deshpande, Sravani Kethireddy, David R. Janero, Mansoor M. Amiji

**Affiliations:** 1 Department of Pharmaceutical Sciences, School of Pharmacy, Bouvé College of Health Sciences, Northeastern University, Boston, Massachusetts, United States of America; 2 Center for Drug Discovery, School of Pharmacy, Bouvé College of Health Sciences, Northeastern University, Boston, Massachusetts, United States of America; 3 Faculty of Pharmacy, King Abdulaziz University, Jeddah, Saudi Arabia; Wake Forest School of Medicine, UNITED STATES

## Abstract

Atherosclerosis and its consequences remain prevalent clinical challenges throughout the world. Initiation and progression of atherosclerosis involves a complex, dynamic interplay among inflammation, hyperlipidemia, and endothelial dysfunction. A multicomponent treatment approach targeted for delivery within diseased vessels could prove beneficial in treating atherosclerosis. This study was undertaken to evaluate the multimodal effects of a novel ω-3-fatty acid-rich, 17-β-estradiol (17-βE)-loaded, CREKA-peptide-modified nanoemulsion system on experimental atherosclerosis. *In vitro* treatment of cultured human aortic endothelial cells (ECs) with the 17-βE-loaded, CREKA-peptide-modified nanoemulsion system increased cellular nitrate/nitrite, indicating improved nitric oxide formation. *In vivo*, systemic administration of this nanoemulsion system to apolipoprotein-E knock out (ApoE^-/-^) mice fed a high-fat diet significantly improved multiple parameters related to the etiology and development of occlusive atherosclerotic vasculopathy: lesion area, circulating plasma lipid levels, and expression of aortic-wall inflammatory markers. These salutary effects were attributed selectively to the 17-βE and/or ω-3 polyunsaturated fatty acid components of the nano-delivery system. At therapeutic doses, the 17-βE-loaded, CREKA-peptide modified nanoemulsion system appeared to be biocompatible in that it elicited no apparent adverse/toxic effects, as indexed by body weight, plasma alanine aminotransferase/aspartate aminotransferase levels, and liver and kidney histopathology. The study demonstrates the therapeutic potential of a novel, 17-βE-loaded, CREKA-peptide-modified nanoemulsion system against atherosclerosis in a multimodal fashion by reducing lesion size, lowering the levels of circulating plasma lipids and decreasing the gene expression of inflammatory markers associated with the disease.

## Introduction

Despite diverse cardiovascular risk-reduction initiatives, pharmacotherapeutic options, and revascularization modalities, cardiovascular disorders primarily due to atherosclerosis (e.g., heart failure, stroke, myocardial infarction) represent persistent unsolved health problems, coronary heart disease being the leading cause of death in the United States [1]. Triggered by vascular injury, atherosclerosis is considered a chronic, lipid-driven inflammatory process characterized by adverse functional deficits and phenotypic changes in vascular endothelial (ECs) and smooth-muscle cells (SMCs) supporting plaque initiation and vessel stenosis [2–4]. Disease progression involves multiple contributors, among which activated endothelium, infiltrating monocytes/macrophages, migrating SMCs, intimal hyperplasia, lipid retention in the vascular wall, and luminal fatty-streak formation are important for generating the primary occlusive lesion, a stable atheromatous plaque [5, 6]. Repeated cycles of inflammation along with dynamic changes in the composition of the plaque microenvironment encompassing mobilized immune cells, an elaborated extracellular matrix, and production of various growth factors/cytokines can promote plaque destabilization and rupture, precipitating thereby an acute thrombotic syndrome with attendant, potentially lifespan-limiting clinical complications [7].

The complex, multifactorial etiology of atherosclerosis and the significant healthcare burden imposed by cardiovascular diseases have fueled the search for improved diagnostic and treatment modalities [8, 9]. To this intent, increasing attention has been paid to the application of nanomedicine for enhancing the mechanism of atherogenesis and diagnosing, preventing, and treating atherosclerosis [10–12]. Nanotechnology-based approaches have advanced the diagnosis of vulnerable atherosclerotic lesions, and nanocarriers have afforded insights into the cellular basis of plaque rupture and the processes of plaque erosion and vessel repair [2, 3]. Experimental demonstration that nanocarriers can be used as drug-delivery devices for reducing clinically important aspects of the atherogenic process, including intimal hyperplasia, suggests the potential therapeutic application of nanotechnology approaches for delivering to the vascular wall one or more bioactive agents whose pharmacological profile suggests potential anti-atherosclerosis benefit [13].

In this regard, the pleiotropic cardio- and vasoprotective properties of naturally occurring, ω-3 polyunsaturated fatty acids (PUFAs) and estrogens have been implicated in the prevention and/or regression of occlusive vascular disease and reduction of associated risk factors. Experimental studies in cellular and animal systems have demonstrated favorable anti-inflammatory, anti-thrombotic, and anti-atherogenic effects of ω-3 PUFAs—especially eicosapentanoic acid [20:5(n-3)] (EPA), docosahexaenoic acid [22:6(n-3)] (DHA), and the essential PUFA α-linolenic acid [18:3(*n*-3)] (ALA)—that include improving endothelial function, reducing platelet activation/aggregation, dampening pro-inflammatory cytokine production and signaling pathways in the arterial wall, and preventing erosion/rupture of unstable atherosclerotic plaque [14, 15]. In humans, plasma ω-3 PUFA levels are directly associated with reduced coronary atherosclerosis progression, and observational epidemiological studies have provided evidence of the health benefits of supplemental ω-3 PUFAs, supporting a clinical lipidology approach for helping manage atherogenic dyslipidemia [16, 17]. Prospective observational studies and adequately powered randomized clinical trials have demonstrated the benefits of supplemental ω-3 PUFAs against coronary heart disease mortality and sudden cardiac death [16, 18]. Clinical trials focusing on the potential benefit of ω-3 PUFAs for primary and secondary prevention in the cardiovascular setting are underway [19].

Supplemental 17-β-estradiol (17-βE) interferes with the progression of coronary atherosclerosis and attenuates inflammation in laboratory models of denudative vascular injury and neointima formation [20] as well as in apolipoprotein E-deficient (apo E^-/-^) mice whose genetically altered lipoprotein metabolism *per se* predisposes to spontaneous atherosclerotic lesion formation [21]. The anti-atherogenic potential of 17-βE has also been suggested by (largely indirect) evidence from observational human studies and clinical trials. Limited results of 17-βE-eluting stents against atherosclerosis have proven equivocal, perhaps reflecting the late 17-βE application in these patients relative to the already significant progression of atherosclerosis and vessel damage [22]. Multiple studies in postmenopausal women support the concept that replacement therapy with 17-βE reduces both the incidence of atherosclerotic coronary artery disease and the risk of cardiovascular mortality, especially when initiated soon after menopause onset and prior to the development of clinical atherosclerosis [4–6]. While oral hormone replacement therapy is not included in the currently recommended therapeutic regimen for atherosclerosis, results from a recent randomized clinical study evaluating the effect of a triphasic hormone replacement therapy with 17-βE in recently postmenopausal women highlight the therapeutic potential of natural estrogens in reducing the risk of mortality associated with cardiovascular disorders with no increase in risk of any cancer or other vascular complications [23]. Well-recognized atheroprotective actions of 17-βE include reducing oxidative stress; improving endothelium-dependent vasodilation and vascular tone; inhibiting inflammation and SMC migration; altering the circulating lipoprotein profile to enhance high density lipoprotein (HDL) and reduce low density lipoprotein (LDL); attenuating the release of vasoconstrictor and chemotactic agents and the expression of endothelial monocyte-adhesion molecules; and increasing the expression of endothelial nitric oxide synthase (eNOS) and the production of the potent vasodilator and anti-thrombotic agent, nitric oxide (NO) [4–6].

A proprietary nanoparticle system has been successfully developed and commercialized as an advanced transdermal 17-βE delivery technology [24]. In a similar manner, a nanomedicine-based approach has the potential to optimize the delivery of lipophilic 17-βE to the vascular wall *in vivo*. These considerations, along with data supporting the vasoprotective properties and (pre)clinical efficacy of ω-3 PUFAs and 17-βE against atherosclerosis, prompted us to design an ω-3-fatty acid-containing, flaxseed oil-based nanoemulsion system encapsulating 17-βE that is rapidly internalized by vascular cells, suggesting the potential therapeutic utility of this 17-βE-loaded formulation against occlusive vascular disease [25]. We subsequently modified that original 17-βE nanodelivery system to include the clot-binding peptide cysteine-arginine-glutamic acid-lysine-alanine (CREKA) [26] as a targeting element with binding selectivity for atherosclerotic plaque [27]. When compared directly to other delivery modes *in vivo*, systemic administration of 17-βE to mice using this CREKA-peptide modified nanoemulsion system afforded optimal 17-βE biodistribution and pharmacokinetics with prolonged 17-βE circulation in the plasma and excellent 17-βE targeting efficiency for heart and aorta [26]. These results suggested that this novel delivery system would have significant therapeutic potential against experimental atherosclerosis. Accordingly, the present study was designed to evaluate the selective delivery of 17-βE to aortic atherosclerotic plaque with this CREKA-peptide-modified nanoemulsion system and profile its *in vivo* biocompatibility and efficacy against atherosclerosis in diet-induced, ApoE^-/-^ mice fed a high-fat diet, a well-recognized murine model of experimental atherosclerosis [28]. Rich in ALA, the flaxseed oil in this nanoemulsion system also affords the opportunity for ω-3 PUFA-related anti-atherosclerosis effects, inviting the potential development of a multimodal nanotechnology paradigm for treating atherosclerosis.

## Materials and Methods

### Ethics statement

All animal experiments were performed in accordance with the National Institutes of Health (NIH) Guide for the Care and Use of Laboratory Animals published by the United States NIH and approved by the animal care and ethics committees of Northeastern University, Boston, Massachusetts, USA.

### Materials

17-βE was purchased from Sigma-Aldrich (St. Louis, MO). Extra-virgin, ω-3-PUFA-containing flaxseed oil was kindly provided by Jedwards International (Quincy, MA). Flaxseed oil contained up to 56% by weight alpha linolenic acid, 14.4% by weight linoleic acid, 20.1% by weight oleic acid, 3.56% by weight stearic acid and 5.08% by weight palimitic acid as described in the specification sheet. Egg phosphatidylcholine (Lipoid^®^ E80) and 1,2-dioleolyl-3-trimethylammonium-propane (DOTAP) was kindly provided by Lipoid GmbH (Ludwigshafen, Germany), and 1,2-distearoyl-*sn*-glycero-3-phosphoethanolamine-*N*-[methoxy- (polyethylene glycol)-2000] (DSPE-PEG_2000_) was from Genzyme (Cambridge, MA). The 5-mer CREKA peptide was synthesized by and purchased from the Tufts University Peptide Synthesis Core Facility (Boston, MA). Human aortic ECs, tissue-culture reagents, and media constituents were purchased from Cell Applications- (Catalog number-304K-05a) (San Diego, CA).

Male C57 BL/6 (C57) and ApoE^-/-^ mice were from Taconic (Germantown, NY). The high fat diet (D12079B) was purchased from Research Diets, Inc. (New Brunswick, NJ) and contained 21% by weight fat and 0.21% by weight cholesterol. The nitrate/nitrite, cholesterol, and triglyceride assay kits were purchased from Cayman Chemicals (Ann Arbor, MI). The Nova Ultra Oil-Red-O staining kit was purchased from IHC World, Inc. (Woodstock, MD). α-actin antibody was purchased from Santa Cruz Biotechnology, Inc. (Dallas, TX). Other reagents for the immunohistochemical staining were purchased from Cell Marque (Rocklin, CA). All other chemicals and solvents were of analytical reagent grade.

### Cell culture system

Primary human aortic endothelial cells (ECs), were cultured in T-75 flasks at a temperature of 37°C in a 5% CO_2_ atmosphere. At 80% confluency, the cells were trypsinized and re-seeded.

### Preparation, characterization, and cellular uptake of the nanoemulsion system

The 17-βE loaded CREKA-peptide-containing nanoemulsion systems were prepared by the microfluidization method previously detailed.[25, 26] The aqueous phase consisted of Lipoid® E80 (2.4%, w/v) and the DSPE-PEG2000-CREKA conjugate (0.3%, w/v), which were dissolved in deionized water by stirring for 20 min. The oil phase consisted of flaxseed oil (20%, w/v) in a glass vial. For the 17-βE loaded nanoemulsion system, 17-βE was dissolved in ethanol and added to the oil phase. The organic solvent was then evaporated under a nitrogen stream. Each phase was heated at 70°C for 5 min and then mixed together. The mixture was then emulsified under conditions (12,500 psi for 30 seconds) using the high-pressure Microfluidizer® M-110EH processor (Microfluidics, Newton, MA) optimal for obtaining nanoemulsions with an average droplet diameter of 100–200 nm. Cargo-free nanoemulsion “blank” rich in ω-3 polyunsaturated fatty acid, but without 17-βE, was likewise prepared according to this protocol. For surface modification of the nanoemulsion droplets, the CREKA peptide was conjugated with DSPE-PEG-2000 through the standard cysteine-maleimide conjugation reaction, as detailed in **S1 File** and **Figure A in S1 File.** The chemical identity of the conjugate was confirmed by 400-MHz ^1^H-NMR spectroscopy (**Figure B in S1 File)**. The resulting nanoemulsions were characterized for droplet size, surface charge, and surface morphology by standard procedures described in **S1 File**.

The uptake of the CREKA-peptide modified nanoemulsion system within the cultured EC’s was analyzed by fluorescence microscopy using a rhodamine-123 encapsulated CREKA-peptide modified nanoemulsion system. The fluorescent dye-rhodamine-123 encapsulating CREKA-peptide modified nanoemulsion system was prepared by microfluidization as described earlier. Uptake by the ECs was evaluated by fluorescence microscopy using an Olympus IX51 microscope (Center Valley, PA). Briefly, the cells were plated in six well plates at a density of ten thousand cells per well. After 30 minutes, 1 hour, 3 hours and 6 hours of treatment with the rhodamine-123 encapsulated nanoemulsions, the cells were washed with HBSS, the cover-slips were mounted over glass slides and observed under the microscope at 10-X magnification.

### Effect of 17-βE on endothelial cell NO production

The effect of 17-βE in solution and loaded in the CREKA-peptide nanoemulsion system on NO production from cultured ECs was quantified as total oxidative NO products (nitrate/nitrite) with a colorimetric assay kit based on the Griess reagent (Cayman Chemical). For the experiment, ECs were plated in T25 flasks at a density of 50,000 cells per flask. The cells were treated with17-βE loaded solution, the CREKA-peptide modified blank nanoemulsion, or the 17-βE loaded CREKA-peptide loaded nanoemulsion system for 24 hours. One T25 flask was used for each treatment group. After treatment, the cells were washed and trypsinized. The cell pellet was collected by centrifugation, re-suspended in PBS, and centrifuged at 30,000 rpm for 30 minutes. Sixty μl of the cellular extract was used for the assay and the nitrate/nitrite levels were measured using the assay kit as per the manufacturer’s instructions. The remaining cellular extract was treated with lysis buffer and protein concentration was determined by the BCA assay. The final nitrate + nitrite concentration was reported as pmol of nitrate/nitrite per μg of total cellular protein.

### Experimental animals

Male ApoE^-/-^ mice (4–6 weeks old) and C57 mice were housed under pathogen-free conditions in Northeastern University’s Division of Laboratory Animal Medicine (DLAM) vivarium facility and provided with standard-diet food and water *ad libitum*. All animals were allowed to acclimatize for a week. The ApoE^-/-^ mice enrolled into the 10 week efficacy study and were then placed on a high-fat diet for 10 weeks. The treatment with blank/17-βE nanoemulsion treatment was started at the beginning of week 7 of diet supplementation, at a dosing frequency of once in 3 days, and continued till the end of the study. The animals received a total of 10 doses (25 μg of 17-βE /dose) until the end of the study. Given the specified fatty acid composition of the flaxseed oil used (*vide supra*) and the 20% w/v nanoemulsion content of flaxseed oil, we estimate that the final ALA administered *in vivo* to be some 11% w/v of the administered dose.

### Experimental animal protocol

ApoE^-/-^ mice were divided into four groups: 1) control animals on the standard high-fat diet without nanoemulsion/17-βE treatment-untreated group; 2) animals fed a high-fat diet and administered intravenously the 17-βE solution once every 3 days 3) animals fed a high-fat diet and administered intravenously the blank nanoemulsion (i.e., without 17-βE) once every 3 days; 4) animals fed a high-fat diet and administered intravenously the 17-βE-loaded nanoemulsion once every 3 days. During the study period, body weight was monitored weekly. At the end of the study period, animals were euthanized using CO_2_ inhalation followed by cervical dislocation, and blood, heart (aortic sinus), aorta, liver, and kidneys were collected for further analysis. During study initiation, mouse whole blood was collected by submandibular bleeding technique while at study termination blood was collected by cardiac puncture into EDTA tubes. Collected blood samples were then centrifuged for 10 minutes at 2000 x g and plasma was collected and stored at -20°C until further analysis.

### Plasma cholesterol and triglyceride measurements

Plasma cholesterol and triglyceride levels were quantified using cholesterol and triglyceride assay kits from Cayman Chemicals, as per the manufacturer’s instructions.

### Histopathological and immunohistochemical analysis of plaque area

Plaque in the aortic sinus was analyzed for its morphology and cellular composition. Sections of aortic sinus were stained with hemaotoxylin and eosin (H&E), Oil-red- O (for lipid), elastin stain, and α-actin antibody (for SMCs). Oil-red-O staining was performed on frozen cut sections, whereas the other staining was performed on formalin fixed, paraffin embedded sections.

#### Tissue sectioning and morphometric analysis of aortic lesions

The freshly isolated heart and aorta of the animals from the different groups, were embedded in the Tissue-Tek OCT compound (Sakura Finetek Inc., Torrance, CA) and stored at -80°C. The samples were then processed at the Tufts Medical Center (Boston, MA) for further analysis. The frozen samples were equilibrated to -20°C and cut into 10 μm sections for Oil-red-O staining using the Microm HM550 cryostat-MICROM International (GmbH, Germany). For the aortic root, starting at the base of the aortic root, the sections were cut serially at 10 μm thickness with 4 sections per slide. The remaining tissues were then thawed to room temperature, washed with PBS to remove any residual OCT and then embedded in paraffin blocks for the other histochemical (H&E and Elastin) and immunohistochemical analysis. For all the other staining procedures, the paraffin embedded tissues were cut into sections at a thickness of 6 μm, placed on pre-cleaned charged microscopic slides and used for the further staining.

#### Oil-red-O staining

Frozen slides were air dried at room temperature for 60 minutes and then fixed in formalin for 15 minutes. Following fixation the slides were rinsed in 3 changes of distilled water and then placed in pre-stain solution for 10 minutes to avoid carrying any water into the Oil Red O solution. In the meantime, the Oil Red O solution was warmed and the slides were then placed in the pre-warmed solution for 15 minutes at 60°C in an oven. After 15 minutes, the slides were immediately placed in a differentiation solution for 10 minutes and rinsed in three changes of tap water. In the final step of this staining, the slides were counter-stained with Mayer’s hematoxylin solution for 1 minute, rinsed using running tap water for 5 minutes and finally placed in distilled water for 5 minutes. The slides were then mounted using the aqueous mounting medium and coverslips, and then digital images were captured using an Olympus IX51 microscope (Chelmsford, MA). The percent of lipid stained area of the lesions was calculated using the Image J (NIH) program.

#### Immuno-staining

The slides containing the paraffin embedded tissue sections were deparaffinized by treating them with Xylene for 5 minutes (two changes), 100% alcohol for 3 minutes (two changes), 95% alcohol for 3 minutes (two changes) and lastly deionized water for 5–7 changes in a sequential fashion. The slides were then treated with 3% hydrogen peroxide for 20 minutes and then thoroughly rinsed in deionized water. The next step was the antigen retrieval step where the slides were placed in a chamber containing the Cell Marque-Declere solution (citrate based: 10 ml in 250 ml of deionized water) and then placed (uncovered) in a pressure cooker with water at the bottom of rack under steam settings for 15 minutes. The slides were then removed and allowed to cool for 20 minutes and then washed in several changes of deionized water. The next step was the blocking step where the slides were blocked using a Cell Marque-background blocker for 20 minutes, Biogenex Avidin blocker for 15 minutes and then the Biogenex biotin blocker for 15 minutes in a sequential fashion. Between each blocking step, the slides were rinsed 2–3 times with PBS. The final stage of the blocking step involved incubating the slides for 20 minutes with the Biogenex Power blocker, following which the slides were not rinsed and just blown clear of the blocker. The sections were then incubated with primary antibody—alpha actin for SMCs; for 1 hour at room temperature and then rinsed with PBS. Following the primary antibody incubation, the Cell Marque Detection System was used for detection and the slides were incubated with the biotinylated link for 20 minutes. The slides were then rinsed with PBS and then the Cell Marque-AEC chromagen was applied and incubated for 5 minutes and then rinsed twice with deionized water and once with PBS and transferred to tap water. The sections were then counterstained in hematoxylin by dipping each slide for 10 dips in the hematoxylin solution and rinsing in tap water. Subsequently, the slides were dipped 10 times in the bluing reagent and rinsed in tap water. Finally, the excess water was blotted off, and the slides were dried either overnight at room temperature or by placing in an oven for 20 minutes. After a quick dip in xylene, the Cell Marque-aqueous mounting media was applied and the coverslip was mounted on the slides and the slides were observed under the microscope. The digital images of the stained specimens were captured using an Olympus IX51 microscope (Chelmsford, MA). The percent of SMC stained area of the lesions was calculated using the Image J (NIH) program.

#### H&E staining

For the hematoxylin and eosin staining the slides were deparafinized as described previously, rinsed in tap water for 1 minute and then placed in the Harris hematoxylin solution for 3 minutes. The slides were then washed using tap water for 3 minutes and treated with 2% glacial acetic acid in 70% alcohol for 30 seconds. Following this treatment, the slides were rinsed using tap water and placed in 1% lithium carbonate solution for 30 seconds and rinsed with tap water. The slides were then placed in 80% ethanol for 1 minute and treated with alcoholic eosin solution for 2 minutes and then dipped 8 times in 95% alcohol, 20 times in 100% alcohol and finally 10 times in xylene. These slides were then mounted using the aqueous mounting medium and coverslip and the digital images were captured using the Olympus IX51 microscope. In addition to the heart and aorta, liver and kidney tissues stored in formalin were stained with H&E as described above for the biocompatibility study. The occluded lesion area (% occlusion) was calculated using the Image J (NIH) program.

#### Elastin staining

Tissue sections were stained for elastin using the modified Verhoeff’s elastic tissue stain. The stain consists of a mixture of 3 solutions, Verhoeff solution 1 –hematoxylin in absolute alcohol (1g in 20ml); Verhoeff solution 2–10% aqueous ferric chloride solution and Verhoeff solution 3—iodine (2g/100ml) and potassium iodide (4g/100ml) in water. The three solutions were mixed together to prepare the Verhoeff staining mixture. The paraffin embedded slides were treated with 1% potassium permanganate for 5 minutes, rinsed with tap water until clear an then treated with 1% oxalic acid for 5 minutes and rinsed in tap water. Following this treatment, the slides were de-paraffinized as described previously and stained in the Verhoeff mixture for 15 minutes. The slides were immediately differentiated using 2% ferric chloride and then placed in tap water to stop the differentiation. The slides were then placed in 95% alcohol for 5 minutes, rinsed in water and counter stained using the Van Gieson solution for a minute. The final step involved rinsing in two changes of 95% alcohol, air drying, placing in xylene and then mounting the sections using an aqueous mounting medium and coverslip. The digital images of the stained specimens were captured using an Olympus IX51 microscope (Chelmsford, MA). The percent of elastin stained area of the lesions was calculated using the Image J (NIH) program.

#### Aortic lesion analysis

As mentioned previously, all aortic lesions were analyzed using the captured images and the Image J (NIH) image processing program. For aortic lesion analysis, the plaque area and the specific composition of the plaque–lipid, elastin and SMC content were measured based on total pixels staining positive for the component of interest. The total plaque area was normalized to the total surface area of the aortic root and reported as % occlusion i.e. percentage of the total surface area measured. The specific composition of the plaque–lipid, elastin and SMC content was normalized to the total plaque area and reported as percentage of plaque area measured. For each study group, data was reported as mean ± S.D. from 4 independent samples and each sample measurement consisted of 4 sections quantified per stain of interest.

### Real-time RT-PCR analysis of genes associated with atherosclerosis

At the end of the 10-week study, aortas of animals from each of the 4 treatment groups were isolated and real-time PCR analysis was performed to analyze aortic expression of 9 atherosclerosis-related genes in these study groups. The main goal was to analyze the effect of the different treatments– 17-βE solution/nanoemulsion and blank nanoemulsion on the aortic expression of these 9 genes in the high-fat diet fed ApoE^-/-^ mice. These genes were selected from a panel of 86 atherosclerosis-related genes screened previously as a pilot study. In the pilot study, a baseline gene expression profiling was performed to study the aortic expression of 86-atherosclerosis-related genes of interest from aortic tissue isolated from the following groups– 1) wild-type C57 mice fed with a normal chow diet, 2) ApoE^-/-^ mice fed with a normal chow diet, and 3) ApoE^-/-^ mice fed with a high-fat diet respectively. The assay was performed using a pre-coated 96 well plate consisting of the Atherosclerosis RT2 Profiler PCR array system from SABiosciences (Catalog number PAMM-038Z) and the Roche-LightCycler® 480 system (Indianapolis, IN). The gene expression profile from the wild-type mice receiving the normal diet served as the baseline to compare the gene expression profile in the aortas of the ApoE^-/-^ mice receiving normal or a high-fat diet. Based on the gene expression profile observed in the pilot study (**S1 Fig**); 9 genes were shortlisted and aortic gene expression was reported in the final study. For both the pilot and the final study, aortas were isolated, homogenized and total RNA was isolated from these homogenates using the RNeasy Fibrous Tissue Mini Kit from Qiagen (Valencia, CA). The quality assessment of the isolated RNA was performed using the NanoDrop 2000 UV-Vis spectrophotometer from Thermo Scientific (Wilmington, DE). cDNA was synthesized using the RT2 first-strand synthesis kit from Qiagen/SABiosciences (Valencia, CA).

### Histopathological and immunohistochemical analysis of liver and kidney

At the end of the study, liver and kidney tissue from animals within each of the four treatment groups was collected for histopathological evaluation. Tissue H&E was performed on formalin fixed, paraffin embedded sections, as described earlier and a blinded histopathological analysis was carried out by Dr. Jerry Lyons, a certified veterinary pathologist at the Tufts University Veterinary School (Grafton, MA).

### Plasma enzymes

At the end of the study, plasma alanine transaminase (ALT) and aspartate transaminase (AST) enzyme levels were quantified from plasma samples from animals (n = 4) within each of the four experimental groups using a kit from Pointe Scientific (Fairlawn, NJ) as per the manufacturer’s instructions.

### Statistical analysis

Data are reported as mean values ± SD or SEM for n ≥ 4 independent samples. Comparison between treatment groups was made using either one- or two-way ANOVA, depending upon group number (Prism^®^ software; GraphPad, La Jolla, CA), followed by Tukey’s or Bonferroni’s multiple-comparisons test.

## Results

### The CREKA-peptide modified nanodelivery system is internalized by human vascular ECs and enhances their NO production

**Fig 1:**
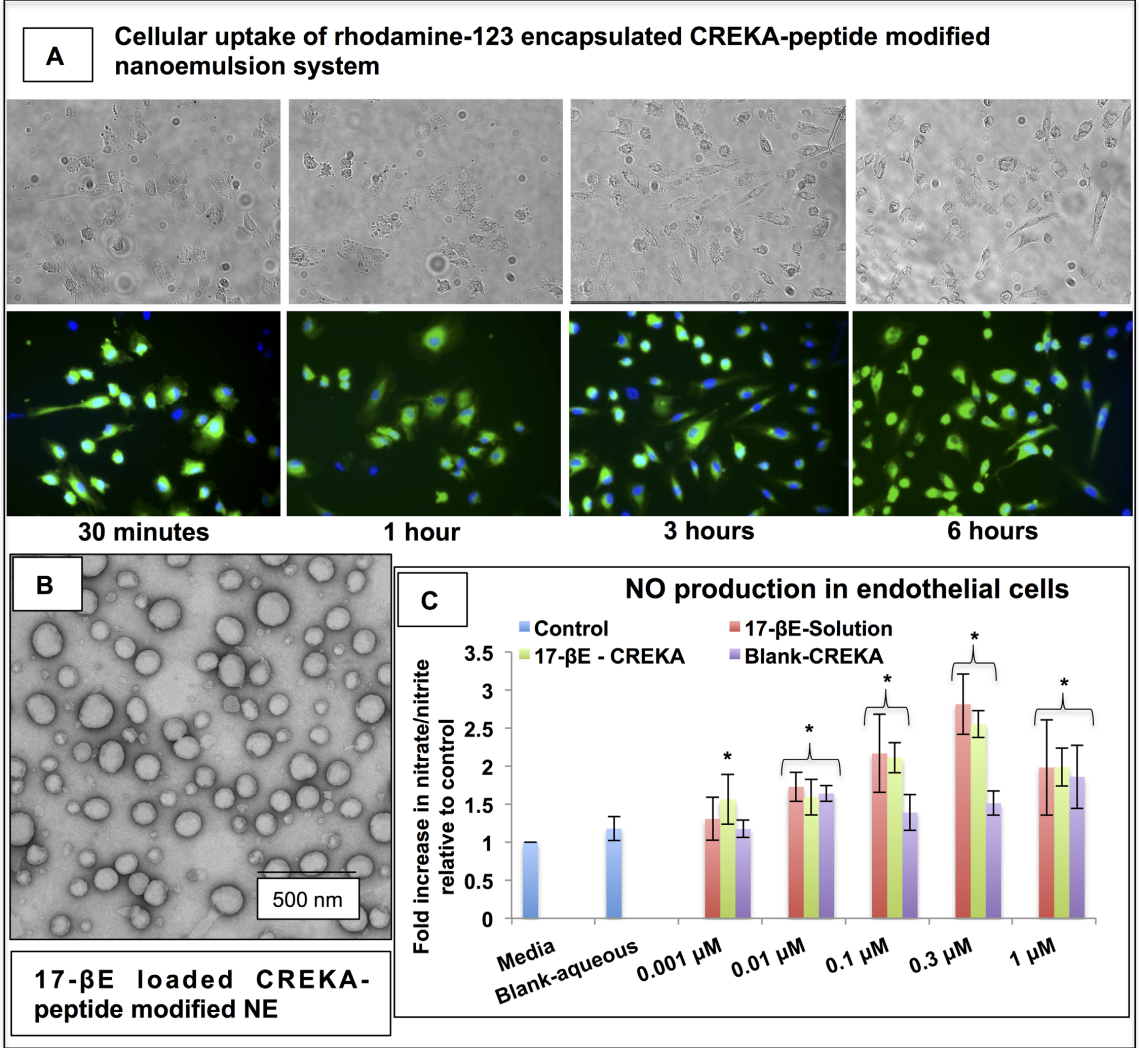
Characterization of the uptake of the 17-βE-loaded CREKA-peptide-modified nanoemulsion system and nitric oxide production by human aortic ECs in culture. (A) Uptake and distribution of the rhodamine-123 dye labeled CREKA-peptide modified nanoemulsion system in cultured human aortic ECs. (B) Transmission electron microscopy of CREKA-peptide modified 17-βE loaded nanoemulsion formulation. The nanoemulsion is spherical with diameter of ~150nm. Scale bar is 500 nm. (C) Effect of 17-βE solution and nanoemulsion on nitric oxide production (nitrate plus nitrite) by cultured human aortic ECs. Data reported as mean ± S.D. for n = 4 independent samples. The asterisk represents statistical significance (*P* < 0.05) between untreated control cells and the different solution and CREKA-peptide-modified nanoemulsion-based treatments indicated.

To establish a precedent for (patho)physiologically-relevant interactions between our CREKA-peptide-targeted nanoemulsion system and the vascular endothelium, we first evaluated by fluorescence microscopy whether the nanoemulsion can deliver encapsulated payload intracellularly using a CREKA-peptide modified nanoemulsion labeled with fluorescent rhodamine-123 dye. This nanoemulsion system was readily internalized by cultured human ECs and successfully delivered the payload within the cells **(Fig 1A).** Although the CREKA peptide binds to fibrin clots, the absence of fibrin meshwork in the EC cultures does not leverage this targeting specificity *in vitro*. Both the “blank” (i.e., not containing 17-βE) CREKA-peptide nanodelivery system and the 17-βE-loaded nanoemulsion both evidenced a comparable average droplet size (187 ± 7.5 nm and 176 ± 4.8 nm, respectively; means ± S.D., n = 3) and surface charge (-54.6 ± 4.1 mV and -56.4 ± 5.1 mV, respectively; means ± S.D., n = 3). The CREKA-peptide, 17-βE-containing nanoemulsion droplets had a uniform, smooth and spherical morphology (**Fig 1B**) with 94.6% encapsulation efficiency at 10% loading of 17-βE.

NO plays a pivotal role in the maintenance of vascular homeostasis, and reduced NO bioavailability has been linked to endothelial dysfunction predisposing to atherogenesis.[29] Since NO has an extremely short half-life and is rapidly oxidized to nitrate and nitrite in cell culture and *in vivo*, we measured the effect of 17-βE in solution and the 17-βE-loaded nanoemulsions on nitrate + nitrite levels in the cell lysates of aortic ECs as an index of their NO production. Treatment of cultured human aortic ECs with blank and 17-βE loaded CREKA-peptide modified nanoemulsion systems, at most concentrations ranging from 0.01 μM to 1 μM, led to a significant increase (p < 0.001) in the nitrate + nitrite produced by the treated ECs relative to untreated cells (media) **(Fig 1C**). At the lowest concentration tested (0.001 μM), 17-βE-CREKA treatment alone led to a significant increase in the nitric oxide levels measured, which may reflect an improved efficacious delivery of the molecule by means of the nanoemulsion system. The blank CREKA-peptide modified nanoemulsion system at 0.01 μM and 1 μM enhanced EC nitrate + nitrite production relative to untreated cells. The increased nitrate + nitrite levels measured on treatment with the blank nanoemulsion was attributed to the ω-3-fatty acid content of the delivery system from the flaxseed oil contained therein. Detailed statistical comparison is reported in **Tables A, B and C in S2 File**.

### Biocompatibility of systemic 17-βE solution and 17-βE-CREKA nanoemulsion

**Fig 2:**
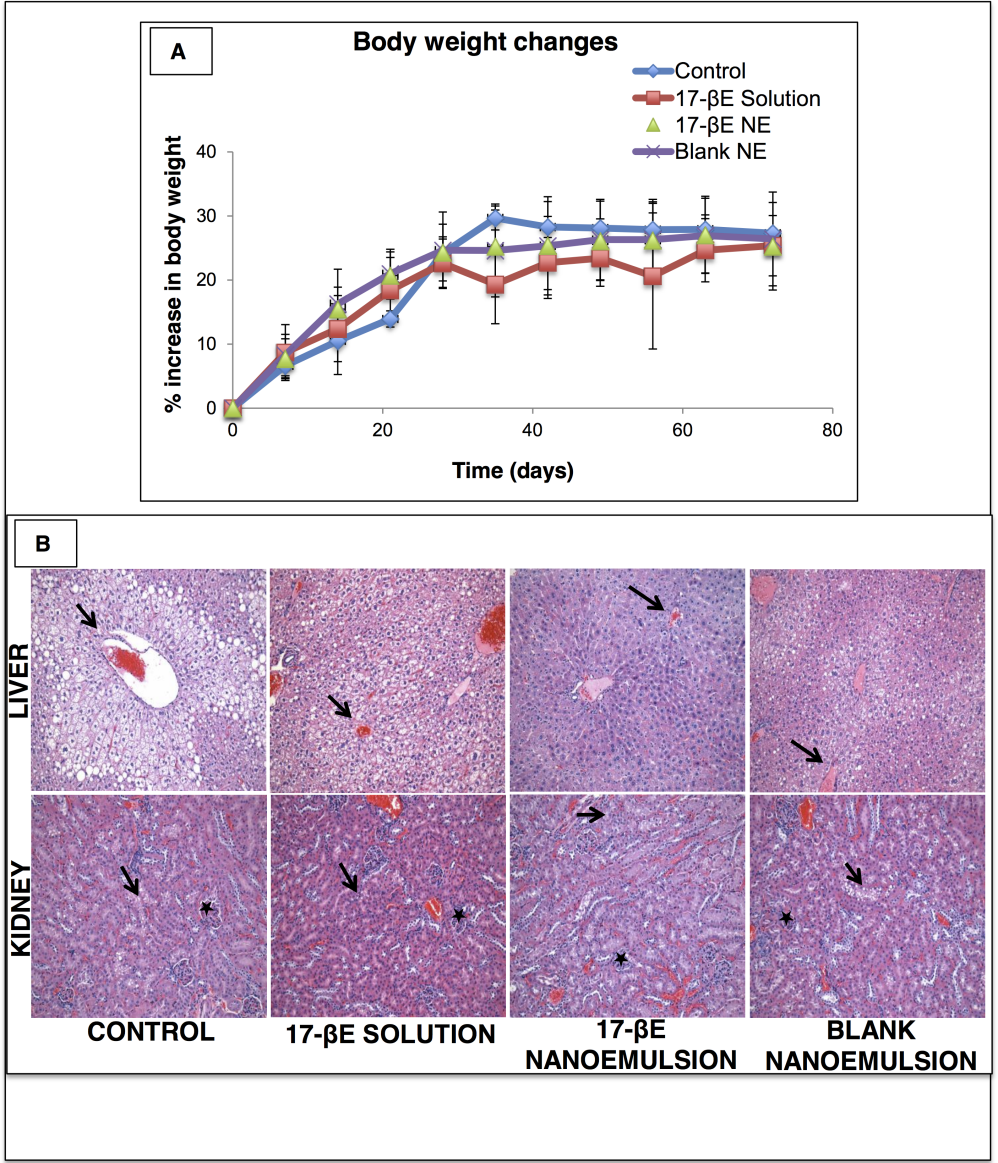
*In vivo* safety profile evaluation of systemic administration of 17-βE as a solution and a CREKA-peptide-modified nanoemulsion system. (A) Body weights of control mice fed a high-fat diet and mice fed a high-fat diet and treated with either the blank nanoemulsion, 17-βE in solution, or the 17-βE-loaded CREKA-peptide-modified nanoemulsion. (n = 8 independent animals per group). (B) Histology of liver and kidney tissues isolated from the control (untreated) and specified treatment groups. (n = 6 independent animals per group).

To evaluate the *in vivo* biocompatibility of repeated systemic administration of 17-βE in solution, the blank CREKA-peptide modified nanoemulsion, and the 17-βE-CREKA-peptide modified nanoemulsion, plasma enzyme markers of hepatic damage (ALT and AST) were measured before and after dosing for 3 weeks (for a total of 10 doses at the end of the study), and body weight was measured weekly. Weight-gain of animals fed a high-fat diet was not affected by treatment with either the 17-βE in solution, the blank nanoemulsion, or the 17-βE-CREKA-peptide modified nanoemulsion treatment examined (**Fig 2A**). The mean body weights measured at study initiation and study termination were similar across the different study groups as reported in **S1 Table**. Similarly, plasma ALT and AST were within the normal range and unaffected by any of these treatments (**Table 1**).

**Table 1:** Plasma aspartate transaminase (AST) and alanine transaminase (ALT).

Treatment Groups	AST Levels (IU/L)		ALT Levels (IU/L)	
	Initial Week*	Final Week*	Initial Week*	Final Week*
No Treatment	32.8 ± 11.8	52.3 ± 13.3	25.5 ± 5.95	61.7 ± 10.86
17-βE in Solution	28.1 ± 5.29	23.3 ± 7.45	22.1 ± 3.63	49.9 ± 9.28
Blank Nanoemulsion	21.0 ± 2.72	23.0 ± 4.29	14.0 ± 0.79	23.5 ± 5.87
17-βE in Nanoemulsion	28.7 ± 6.93	25.6 ± 1.98	15.0 ± 1.17	19.1 ± 2.27

*Data are expressed as the mean ± SEM (n = 6 independent animals per group).

Liver histopathology revealed diffuse periacinar and hepatocellular vacuolation in tissue samples from untreated control animals as well as from animals administered either blank nanoemulsion or 17-βE either in solution or in the 17-βE-CREKA formulations. Hepatocellular vacuolation in liver tissue indicated lipid hepatopathy, and kidney tissue showed lipid deposition within renal tubular cells and moderate multifocal renal tubular vacuolation. (**Fig 2B**). Since these phenomena were apparent in control animals untreated with any solution/nanoemulsion and have been associated with a high-fat diet, we ascribe them to our dietary regimen.

In aggregate, these data demonstrate that the systemic 17-βE in solution, the blank CREKA-peptide nanoemulsion system, or the 17-βE-CREKA nanoemulsion system were well tolerated by ApoE^-/-^ mice and did not induce any apparent organ damage.

### The 17-βE-CREKA nanoemulsion reduces atherosclerotic plaque size

**Fig 3:**
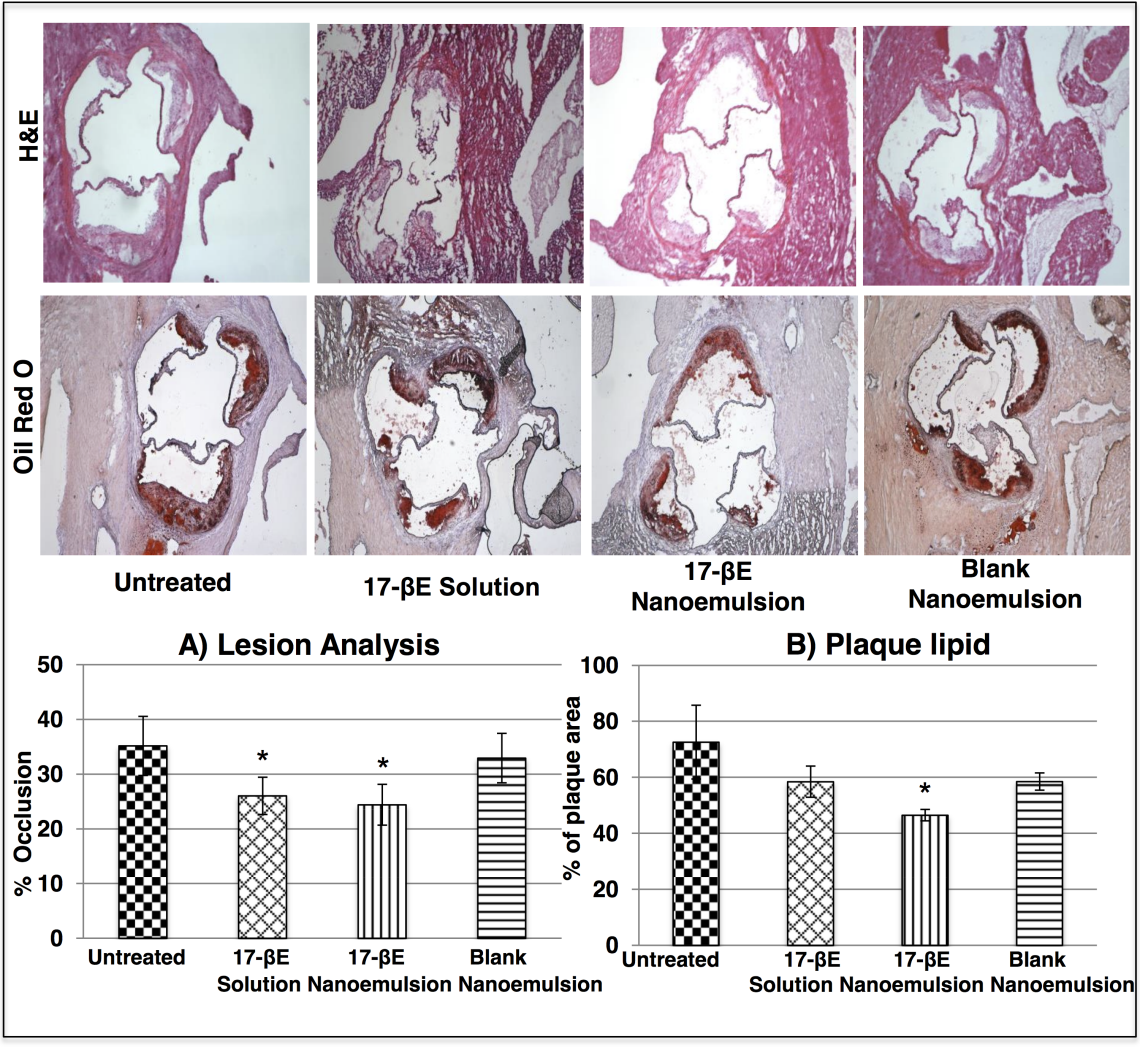
*In vivo* histological evaluation of systemic administration of 17-βE as a solution and a CREKA-peptide-modified nanoemulsion system on plaque size and lipid content. (A) H & E staining of the aortic valves and plaque area analysis quantified using Image J (n = 4 independent animals per group). The asterisk represents statistical significance between the untreated treatment group and the different 17-βE solution, 17-βE nanoemulsion and blank nanoemulsion treatment groups (*P* < 0.05). (B) Oil-red-O staining of the aortic valves and plaque lipid analysis quantified using Image J (n = 4 independent animals per group). The asterisk represents statistical significance between the untreated treatment group and the different 17-βE solution, 17-βE nanoemulsion and blank nanoemulsion treatment groups (*P* < 0.05).

ApoE^-/-^ mice receiving a high fat diet for 10 weeks showed characteristic, early-stage occlusive atherosclerotic lesions within their aortic valves. Differential staining with Oil-O-red, elastin stain, and α-actin antibody indicated that the lesions were comprised of large lipid deposits, a thickened elastin layer, and a small population of migrating SMCs. ApoE^-/-^ mice fed a high-fat diet and no other treatment evidenced ~ 30–35% aortic-valve occlusion, as did animals receiving the blank CREKA nanoemulsion system (**Fig 3**). Treatment with 17-βE in either solution or in the CREKA-peptide modified nanoemulsion significantly reduced the extent of lesion occlusion, whereas the blank CREKA-peptide modified nanoemulsion system had no effect on lesion size. The plaque area measured from the 17-βE loaded nanoemulsion group was significantly different from the plaque area measured from the aortic valves of animals from the untreated group (p < 0.05). Detailed statistical comparison of the plaque area measured across the different treatment groups is reported in **Table A in S3 File**.

**Fig 4:**
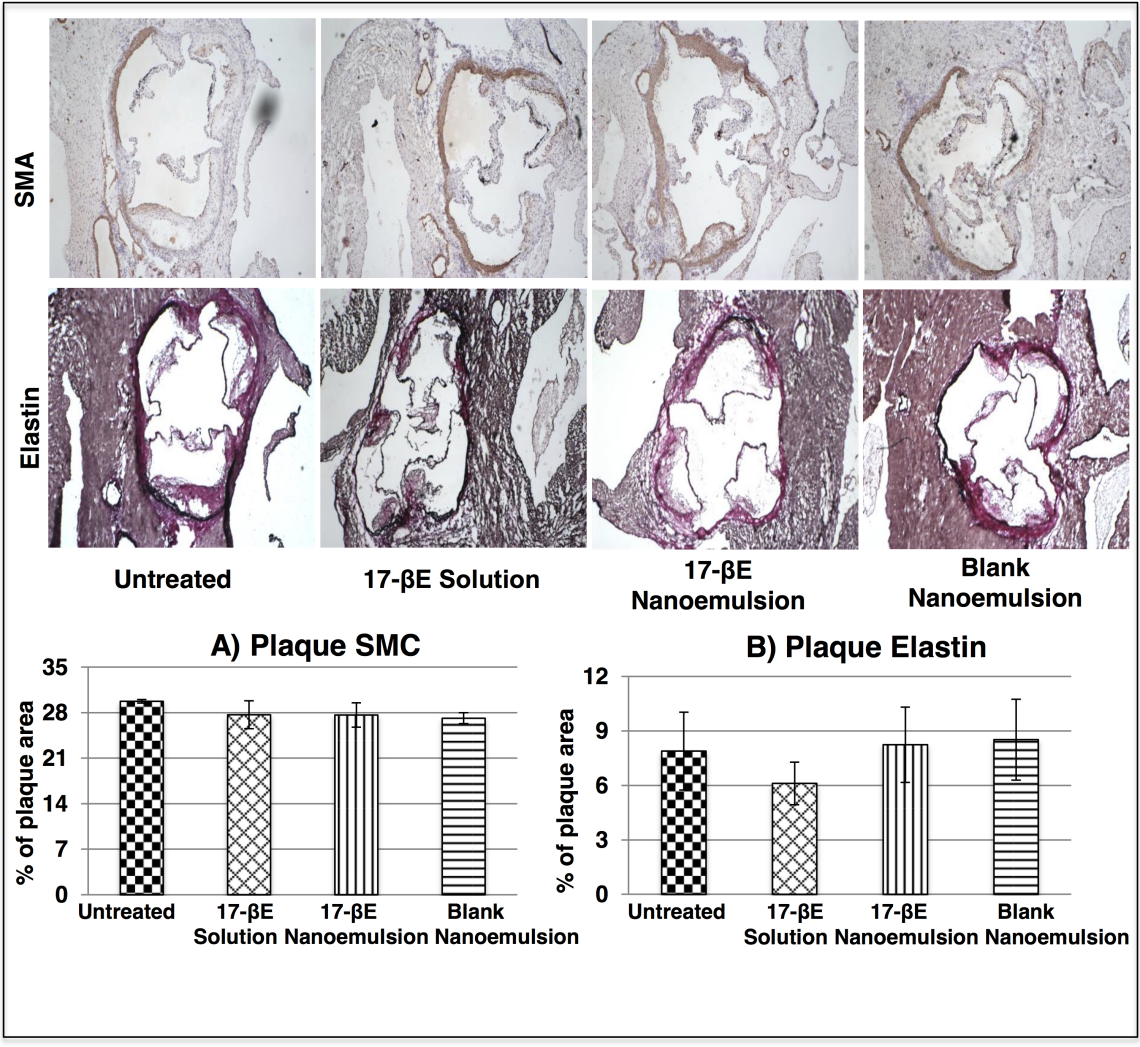
*In vivo* histological evaluation of systemic administration of 17-βE as a solution and a CREKA-peptide-modified nanoemulsion system on plaque elastin and smooth muscle cell content (A) Elastin staining of the aortic valves and plaque elastin analysis quantified using Image J (n = 4 independent animals per group). There was no significant difference in the elastin content across treatment groups. (B) SMA staining of the aortic valves and plaque SMC content analysis quantified using Image J (n = 4 independent animals per group). There was no significant difference in the elastin content across treatment groups.

Further histological evaluation of plaque burden based on specific stains revealed that treatment with 17-βE in the CREKA nanoemulsion form significantly decreased plaque lipid content, a difference that did not reach statistical significance with the 17-βE solution and the blank nanoemulsiuon system (**Fig 3**). Aortic valves from all the experimental groups showed similar elastin contents and populations of migrating SMCs within the plaque (**Fig 4**). The detailed statistical comparison of the lipid content, elastin content, and SMC content measured across the different groups is reported in **Tables B, C and D in the S3 File** respectively.

### The 17-βE-CREKA nanoemulsion alters the expression of atherosclerosis-related genes within aorta and improves plasma lipid profile

**Fig 5:**
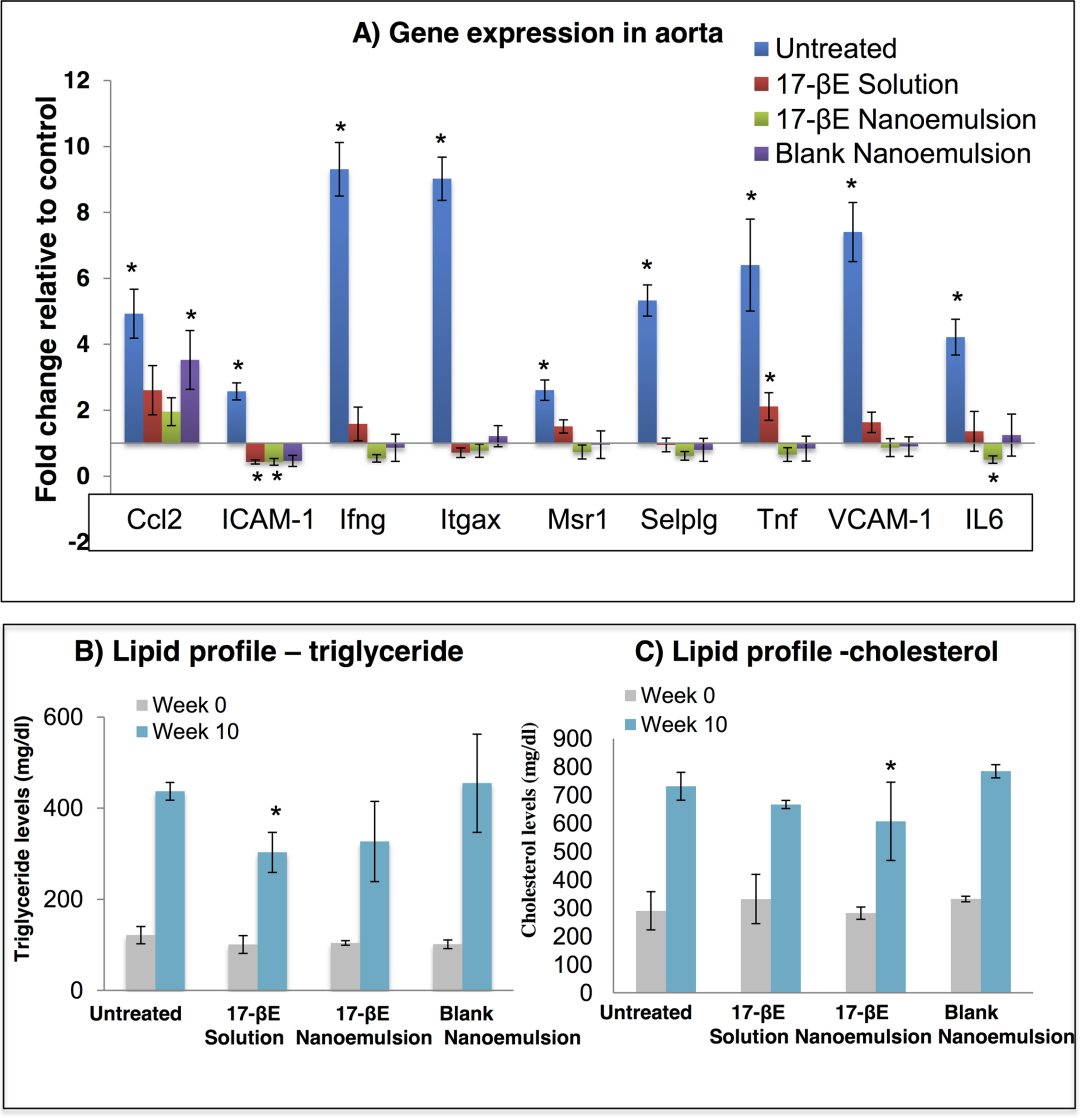
Systemic administration of 17-βE as a solution and a CREKA-peptide-modified nanoemulsion system alters atherosclerosis-related gene expression within the aorta and improves plasma lipid profile. (A) The effect of 17-βE in solution, 17-βE in nanoemulsion, and the blank nanoemulsion on relative gene expression in ApoE^-/-^ mice receiving a high-fat diet measured relative to gene expression in wild-type mice. The asterisk indicates statistical significance (*P* < 0.05) relative to the baseline gene expression measured in the wild-type mice. (n = 4 independent animals per group). (B) Total plasma cholesterol levels measured in the untreated and the different treatment groups at the beginning and end of the efficacy study. Values are represented as means ± SD (n = 6 independent animals per group). The asterisk represents significance between the untreated animals and the different treatment groups (*P* < 0.05). (C) Total plasma triglyceride levels measured in the untreated and the different treatment groups at the beginning and end of the efficacy study. Values are represented as means ± SD (n = 6 independent animals per group). The asterisk represents significance between the untreated animals and the different treatment groups (*P* < 0.05).

We first screened the expression of 86 atherosclerosis-related genes using RNA isolated from aortas of wild-type C57 mice fed a standard diet, ApoE^-/-^ mice receiving a standard chow diet, and ApoE^-/-^ mice receiving a high-fat diet (**S1 Fig.**) as a pilot study. For the final efficacy evaluation, we selected a panel of 9 genes from the pilot study and analyzed the effect of the different treatments– 17-βE solution/nanoemulsion and blank nanoemulsion on the aortic expression of these 9 genes in the high-fat diet fed ApoE^-/-^ mice. Relative to the expression profile of wild-type C57 mice that served as baseline comparator, the aortic wall of ApoE^-/-^ mice receiving a high fat diet (untreated group) showed a significant upregulation of genes for the adhesion molecules Ccl2 [Chemokine (C-C motif) ligand 2], ICAM-1 (intercellular adhesion molecule-1), Itgx (integrin alpha X), Selplg [selectin, platelet (p-selectin) ligand] and VCAM-1 (vascular cell adhesion molecule-1) and for the inflammatory markers Ifng (interferon gamma), Msr1 (macrophage scavenger receptor-1), Tnf (tumor necrosis factor) and IL6 (interleukin 6) (**Fig 5A**). Systemic administration of 17-βE in solution to the high fat diet fed ApoE^-/-^ mice showed a marginal increase in the expression of the genes Ccl2, Ifng, Msr1, Tnf, VCAM-1 and IL6 and decrease in the expression of ICAM-1, Itgax and Selplg, respectively, as compared to the wild type mice. Overall, the treatment with 17-βE solution showed a much lower expression of the pro-atherosclerotic markers as compared to the animals from the untreated group.

Accordingly, the potential influence of systemic administration of the blank and the 17-βE loaded CREKA-peptide modified nanoemulsion systems on the expression of these 9 genes within the aortic wall was next determined. Treatment of the high-fat diet fed ApoE^-/-^ mice with either 17-βE nanoemulsion or blank nanoemulsion induced either a similar or a significant downregulation of the pro-atherosclerotic genes relative to the age-matched wild type mice. Overall, treatment of the high-fat diet fed ApoE^-/-^ mice with either the 17-βE nanoemulsion or blank nanoemulsion led to a decrease in the expression of the atherosclerotic markers within the aortic wall of these animals as compared to the high-fat diet fed ApoE^-/-^ mice with no treatment. These results suggested that treatment with 17-βE loaded CREKA-peptide modified nanoemulsion or with the blank nanoemulsion alone can thus help reduce the atherosclerotic plaque burden by down-regulating the expression of genes that promote atherosclerosis in the ApoE^-/-^ mice. The altered gene expression on treatment with the blank nanoemulsion was attributed to the ω-3-fatty acid content of the delivery system. Detailed statistical evaluation and fold differences have been reported in **Table A in S4 File**.

Due to the high-fat diet, the plasma triglycerides and cholesterol increased significantly over the 10-week study period. Treatment of ApoE^-/-^ mice with 17-βE in solution decreased plasma triglyceride, and treatment of ApoE^-/-^ mice with 17-βE -CREKA nanoemulsion marginally decreased plasma cholesterol levels alone relative to their respective levels in the plasma of untreated animals (**Fig 5B and 5C**). The blank nanoemulsion system did not affect the plasma lipid profile of ApoE^-/-^ mice fed a high-fat diet (**Fig 5B and 5C**). Detailed statistical analysis is given in the **Tables B and C in S4 File**.

## Discussion

The past decade has seen increasing interest regarding the potential application of nanotechnology to atherosclerosis diagnostics and therapy.[2, 12, 30] In this study, we have evaluated a novel nanotechnology platform delivering 17-βE to atherosclerotic lesions *in vivo* for its effect on several parameters of experimental atherosclerosis. The cellular data demonstrate that the 17-βE payload in our CREKA-peptide-modified nanoemulsion system is efficiently taken up by cultured human aortic ECs to potentiate cellular NO production. 17-βE is known to induce an increase in cellular NO production by modulating the function of eNOS. The improved eNOS function is induced as a rapid response through a plasma membrane-associated estrogen receptor (ER)alpha, that activates multiple non-genomic, non-nuclear signaling cascades like the PI3-kinase/Akt signaling pathway and thereby induce eNOS and potentiate endothelial NO production. [31,32] The other mechanism involved in improving eNOS function is a slower response that involves activation of cytosolic ER and its translocation into the nucleus resulting in a genomically regulated increase of eNOS mRNA and protein expression. [33] In addition, several other pathways are involved in the increased bioavailability of NO, which includes reduced oxidative degeneration of NO through suppression of oxidative stress by 17-βE. [33] Early atherosclerosis is associated with impaired endothelial nitric oxide synthase (eNOS) activity, which along with any associated oxidative/nitrosative stress, compromises NO bioavailability.[34] Among the most potent vasodilators and anti-platelet agents, NO from the vascular endothelium contributes to blood-vessel health in multiple ways, e.g., by helping maintain vascular tone and compliance and preventing pro-inflammatory interactions between the endothelium and blood elements to maintain an antithrombotic state.[35, 36] Functional or quantitative compromise of endothelial NO generation undermines the homeostatic actions of NO on the vessel wall and is a key factor in initiating local inflammation and thrombus formation that can lead to atherosclerosis and its clinical sequellae. Thus, employing strategies like the 17-βE loaded CREKA-peptide modified nanoemulsion system that improve/increase NO bioavailability in the vasculature might prove beneficial in the treatment of atherosclerosis.

Our previous pharmacokinetic study of the 17-βE loaded CREKA-peptide modified nanoemulsion system demonstrated its targeting efficiency to the vascular wall and the pronounced accumulation of nanoemulsion-derived 17-βE both locally (heart and aorta) and systemically (plasma) [37]. Those data constitute a strong rationale for the evaluation in our current study of the potential therapeutic efficacy of the 17-βE loaded CREKA-peptide modified nanoemulsion system against experimental atherosclerosis in ApoE^-/-^ mice. The present *in vivo* results demonstrate that treatment of ApoE^-/-^ mice with the 17-βE loaded CREKA-peptide modified nanoemulsion: 1) reduced luminal occlusion within the aortic valves and reduced lesion lipid content; 2) marginally reduced the levels of circulating lipids (mainly cholesterol); 3) downregulated the gene expression for several key pro-inflammatory markers across the aortic wall; 4) was devoid of renal or hepatotoxicity at therapeutically efficacious doses. A high-fat diet accelerates the lesion development in ApoE^-/-^ mice.[28] We observed that after 10 weeks, our high-fat diet produced fibro-proliferative lesions within the aortic valve of ApoE^-/-^ mice, as shown by Oil-red-O staining (**Fig 3**). These lesions denote a relatively early stage of atherosclerosis that mainly involves foam cell formation and a small population of migrating SMCs (**Fig 4**).[38] Treatment with 17-βE solution and 17-βE-loaded, CREKA-peptide modified nanoemulsion system significantly reduced the aortic valve occlusion and, marginally, lesion lipid content. Systemically, the solution and the nanoemulsion system induced a marginal reduction in the plasma triglyceride and cholesterol levels, respectively. However, there was no effect observed on the lesion SMC population or elastin layer.

Human epidemiological studies have highlighted the lipid-lowering effects of estrogens, and 17-βE is known to inhibit cholesterol accumulation, modulate hepatic lipid metabolism, increase the circulation of vasoprotective HDL, and prevent the LDL-oxidation through estrogen-receptor (in)dependent mechanisms.[4] However, in the ApoE^-/-^ mice, circulating levels of cholesterol and triglycerides do not correlate with the extent of atherosclerosis development, i.e., lesion size and area.[21] Thus, while the marginal reduction in the circulating lipids (cholesterol and triglycerides) observed with the 17-βE solution and nanoemulsion treatment might contribute to anti-atherosclerotic vasoprotection, the lesion-size reduction we have observed with the 17-βE solution and nanoemulsion treatment is more likely attributable to effects of 17-βE on parameters other than the circulating lipid profile.

During the early stages of atherosclerosis, SMCs are mainly involved in upregulation of adhesion molecules and pro-inflammatory cytokines that promote atherogenesis.[39] Our nano-delivery system significantly downregulates the gene expression of atherogenic adhesion molecules and inflammatory cytokines. Atherosclerosis is considered an inflammatory disorder whose progression depends upon both vascular and circulating immune cells.[38] Several pro-inflammatory molecules are considered markers for monitoring clinically the development of atherosclerosis.[40] Throughout all the stages of the disease, monocyte attachment to the endothelial surface and migration into the sub-endothelial region constitutes an important component of the inflammatory cascade.[41] Of the inflammatory biomarkers investigated in our study, Ccl2 plays a key role in monocyte infiltration within the sub-endothelial layer of the aorta and has a significant expression within human plaques as well.[42, 43] The adhesion molecules ICAM-1, Itgax, VCAM-1 and Selplg have been studied extensively and are associated with initiation and progression of atherosclerosis. ICAM-1 is mainly expressed across the endothelial surface and plays an important role in the early stages of the disease, inducing the adhesion of lymphocytes, monocytes and neutrophils to the endothelial surface further leading to their transmigration within the aortic intimal layer.[44] Similarly, Itgax promotes atherosclerosis associated with elevated cholesterol levels through activation and adhesion of circulating monocytes and their differentiation into foamy monocytes that adhere to the ECs and are further deposited within the arterial lumen with the help of VCAM-1.[45] Whereas VCAM-1 is mainly associated with firm adhesion of monocytes to the arterial wall,[46] Selplg is a member of the selectin family initiating leukocyte rolling and leucocytes-endothelium, platelets-endothelium and leucocytes-platelet interactions.[47] Lastly, inflammatory markers Ifng and Tnf, are actively involved in the inflammatory cascade associated with atherosclerosis,[48, 49] and the initial and intermediate stages of atherosclerosis also involve an up-regulation of the Msr-1 gene associated with the uptake of LDL cholesterol by macrophages within the arterial wall.[50, 51] In the current study, the ApoE^-/-^ mice fed a high-fat diet showed significant upregulation of these atherosclerosis biomarkers, a pro-atherogenic response attenuated across the aortic wall by treatment with 17-βE solution, 17-βE loaded CREKA-peptide modified nanoemulsion system as well as the “blank” (i.e., without 17-βE) CREKA-peptide modified nanoemulsion system (**Fig 5**). Thus, our results suggest that the atheroprotective effects of the 17-βE solution, 17-βE loaded CREKA-peptide modified nanoemulsion system and blank CREKA-peptide modified nanoemulsion involve modulation of the vascular microenvironment through interaction with the variety of cell types across the aortic valve, including the activated endothelium, migrating SMCs and the infiltrating immune cell population. One plausible mechanism, not currently investigated could involve the decrease of oxidative stress within the plaque microenvironment by the 17-βE solution, 17-βE loaded CREKA-peptide modified nanoemulsion system and blank CREKA-peptide modified nanoemulsion, through downregulation of Nox1 and Nox4 NADPH oxidases that are known for their role in the progression of atherosclerosis in ApoE^-/-^ mice.[52, 53]

Another key finding from our study is the downregulation of the inflammatory-marker gene expression induced by the blank CREKA-peptide modified nanoemulsion. This effect was attributed to the flaxseed oil in the nanoemulsion, a rich source of bioactive ALA. Dietary supplementation with flaxseed leads to a significant regression of atherosclerotic plaque in hypercholesterolemic rabbits [54, 55] and in the cholesterol-fed, low density lipoprotein (LDL) receptor deficient mice through its anti-inflammatory and anti-proliferative effects [56]. Similar results from experimental studies, clinical trials and epidemiological data have highlighted the various cardioprotective effects of flaxseed and its ALA content, which include mitigating the progression of atherosclerosis [57, 58]. While the metabolic fate of ALNA involves a series of alternating desaturation and elongation reactions, where EPA and DHA are the intermediate metabolite and the final product respectively, dietary intervention studies have demonstrated that humans and rodents have a lower efficiency of converting ALA to EPA and DHA [15]. Although the EPA and DHA levels were not measured in the current study, our results suggest that ALA might exert its atheroprotective effects independent of EPA and DHA, warranting further investigation of these independent mechanisms of actions. Thus, along with the salutary benefits attributable to 17-βE delivery, the distinct improvement of vascular status induced by the blank nanoemulsion in atherosclerotic ApoE^-/-^ mice indicates that our novel nanaoemulsion system may have potential as a multimodal approach for treating occlusive vascular syndromes. This concept gains particular significance from the increasing exploitation of polypharmacology and leveraging of the pleiotropic effects of known natural substances/drugs for improved therapeutic outcome of complex cardiometabolic diseases such as diabetes and atherosclerosis.[59] Although estrogen therapy carries the risk of dose-dependent feminizing effects in males, largely observational data support the proposition that estrogen insufficiency/reduced estrogenic action is a risk factor for cardiovascular disease in aging men [60], for whom low-dose estrogen supplementation can exert cardioprotective effects [61]. This context invites future studies incorporating non-feminizing estrogen(-like) compounds into our novel nanoemulsion platform, some of which are indeed being explored as candidate therapy for atherosclerosis (and other) diseases [62].

Development of atherosclerosis in humans is a chronic process over several years, if not decades.[2] Thus, most non-invasive treatment options must belong-term in nature. In our current study, we have determined the effect of a short-term 17-βE treatment (21-day administration) delivered in a ω-3 PUFA-containing nanoemulsion system on early vascular lesions with only 30% vessel stenosis. Evaluating the effect of long-term 17-βE administration in animals with advanced stages of atherosclerosis (60–70% stenosed valves with fibrous cap or calcified lesions) is warranted to characterize further the potential therapeutic efficacy of our 17-βE-loaded CREKA-peptide-modified nanoemulsion system in treating atherosclerosis. Nonetheless, as demonstrated in this study, systemic administration of 17-βE loaded CREKA-peptide-modified-nanoemulsion system can reduce pathological contributors to early atherosclerosis in a well-accepted experimental model of occlusive vascular disease without signs of systemic adversity (i.e., weight-loss) or organ damage. More generally, this nanoemulsion system would appear to have potential as a suitable platform for delivering a variety of therapeutic agents to the vascular wall for addressing atherosclerosis.

## Supporting Information

S1 Table Body weight measurements at study initiation and study termination. (DOCX)

S1 Fig Gene expression profile-clustergram of 86-atherosclerosis-related genes. (DOCX)

S1 File Standard procedure for chemical conjugation reaction i.e. cysteine-maleimide conjugation reaction (Figure A in S1 File) and standard procedure for characterization of the synthesized conjugates (Figure B in S1 File) and the nanoemulsion system. (DOCX)

S2 File Statistical comparison (ANOVA) of nitrate/nitrite levels measured across different treatment groups. **Table A in S2** F**ile** reports comparison between no treatment, blank aqueous treatment and 17-βE solution treatment groups; **Table B in S2 File** reports comparison between no treatment, blank CREKA-peptide modified nanoemulsion and 17-βE loaded CREKA-peptide modified nanoemulsion treatment groups; and **Table C in S2 File** reports comparison between blank CREKA-peptide modified nanoemulsion, 17-βE loaded CREKA-peptide modified nanoemulsion and 17-βE loaded solution treatment groups respectively.
(DOCX)

S3 File Statistical comparison (ANOVA) of lesions area and lesion content analysis. **Table A in S3 File** reports comparison of lesion area measured across the different study groups; **Table B in S3** F**ile** reports plaque lipid area measured across the different study groups; **Table C in S3 File** reports plaque SMC-actin stained area measured across the different study groups and **Table D in S3 File** reports plaque elastin stained area measured across the different study groups respectively.
(DOCX)

S4 File Detailed statistical analysis of the effect of the 17-βE-CREKA nanoemulsion on the expression of atherosclerosis-related genes within aorta and circulating plasma lipids. **Table A in S4 File** represents fold change values and statistical analysis of gene expression in apoE^-/-^ mice receiving high fat diet and the 17-βE solution, 17-βE nanoemulsion and blank nanoemulsion treatment groups measured relative to gene expression in wild type mice. **Table B in S4 File** represents statistical analysis of total plasma triglycerides measured across the different study groups and **Table C in S4 File** represents statistical analysis of total plasma cholesterol measured across the different study groups.
(DOCX)
