# Supplementary material for: Therapeutic Efficacy of an ω-3-Fatty Acid-Containing 17-β Estradiol Nano-Delivery System against Experimental Atherosclerosis
Source: PLoS One. 2016 Feb 3;11(2):e0147337. doi: 10.1371/journal.pone.0147337 (PMC4740455; doi:10.1371/journal.pone.0147337)
Supplement: S1 Table — (DOCX) [file pone.0147337.s001.docx]

**S1 Table: Body weight measurements at study initiation and study termination**

| **Treatment Groups** | **B.W. measurement (grams)** | |
| --- | --- | --- |
|  | **Initial Week*** | **Final Week*** |
| No Treatment | 23.95 + 0.37 | 32.95 + 0.48 |
| 17-βE in Solution | 25.43 + 2.38 | 33.86 + 3.48 |
| 17-βE in Nanoemulsion | 23.81 + 2.15 | 31.99 + 2.90 |
| Blank Nanoemulsion | 22.93 + 1.78 | 31.49 + 2.76 |

*Data are expressed as the mean ± S.D. (n = 8 independent animals per group).
