# Supplementary figures and images for: Therapeutic Efficacy of an ω-3-Fatty Acid-Containing 17-β Estradiol Nano-Delivery System against Experimental Atherosclerosis
Source: PLoS One. 2016 Feb 3;11(2):e0147337. doi: 10.1371/journal.pone.0147337 (PMC4740455; doi:10.1371/journal.pone.0147337)

**S1 Figure:** Gene expression profile-clustergram of 86-atherosclerosis-related genes.


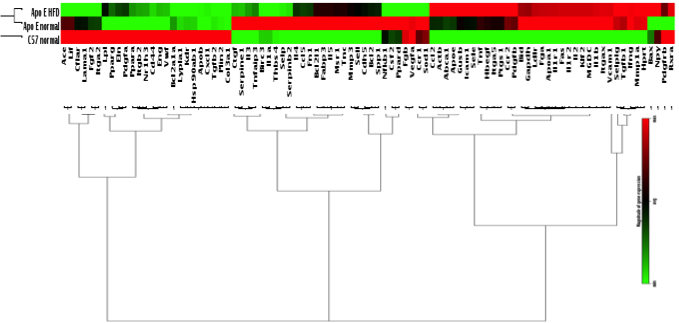

Supplement: S1 Fig — (DOCX) [file pone.0147337.s002.docx]
