# Supplementary material for: Therapeutic Efficacy of an ω-3-Fatty Acid-Containing 17-β Estradiol Nano-Delivery System against Experimental Atherosclerosis
Source: PLoS One. 2016 Feb 3;11(2):e0147337. doi: 10.1371/journal.pone.0147337 (PMC4740455; doi:10.1371/journal.pone.0147337)
Supplement: S1 File — (DOCX) [file pone.0147337.s003.docx]

**Supporting Information**

**S1 File: Standard procedure for chemical conjugation reaction i.e. cysteine-maleimide conjugation reaction (Figure A) and standard procedure for characterization of the synthesized conjugates (Figure B) and the nanoemulsion system**

**1. Cysteine-maleimide conjugation reaction**

For surface modification of the nanoemulsion droplets, the CREKA-peptide was conjugated with the DSPE-PEG-2000 through the cysteine-maleimide conjugation reaction (**S1 Fig.**) and this conjugate was incorporated in the aqueous phase of the nanoemulsion system as described earlier. The DSPE-PEG-CREKA conjugate was synthesized by dissolving 0.1 g of DSPE-PEG2000 Maleimide (mwt 2941.605, 1.36 mmol) in 25 ml PBS (pH 7.4) and mixed well until a clear solution was formed. At pH below 8, the maleimide group serves as an excellent substrate for selective thiol modification, forming a thioether conjugate. To this solution, a two-fold molar excess (0.042 g) of the CREKA peptide (mwt 605.71, 2.72 mmol) was added, the dissolved oxygen was displaced under a nitrogen stream, and the reaction was then sealed and stirred overnight at 4^o^C. The mixture was then dialyzed thoroughly using a cellulose dialysis membrane (MW cut off ~ 2000) for 10 hours to remove excess unconjugated peptide. Following dialysis, the final product was lyophilized and stored at -20^o^C. The conjugation was verified by 400 MHz 1H-NMR spectroscopy (Varian, Inc. CA). About 3 mg of the conjugate was dissolved in 600 μL D20 for NMR analysis (**S2 Fig.**).

**2. Characterization of the nanoemulsion formulation**

Nanoemulsion formulations were characterized with respect to size, surface charge, and surface morphology as reported[^1^](#_ENREF_1) and described below

**2.1 Particle size**

For average droplet size and size-distribution analysis, the Zetasizer (Malvern, Worcestershire, UK) instrument was used. Blank and drug-loaded CREKA-peptide-modified nanoemulsions were diluted (1:10,000, v/v) with deionized water for both droplet size and surface charge measurements. The average droplet size was determined based upon the dynamic light scattering observed at room temperature and a 90^o^ fixed angle. The average hydrodynamic diameter and the polydisperisty index were determined at a count rate of 50-500 kcps to ensure reproducibility.

**2.2 Surface charge**.

For surface charge measurement, samples of diluted nanoemulsion systems were placed in an electrophoretic cell with the refractive index set at 1.33 and the viscosity at 1 cps. Charge was determined based on the electrophoretic mobility of the nanoemulsion droplets, and the zeta potential was calculated using the Smoluchowski equation.[^2^](#_ENREF_2) For both droplet size and surface charge, results were calculated as the average of four samples and expressed as means + SD.

**2.3** **Surface morphology**

For surface morphology analysis, samples were analyzed using transmission electron microscopy. Briefly, samples were loaded onto 200-mesh Formvar-coated copper grids (Electron Microscopy Science, Hatfield, PA), negatively stained with uranyl acetate and dried. Grids were loaded onto the sample holder for visualization with a JEOL 100-X transmission electron microscope (Peabody, MA).

**2.4** **Drug loading efficiency**

The loading efficiency of the 17-βE-loaded, CREKA-peptide-modified nanoemulsions was determined using a high-performance liquid chromatography (LC) assay. The analytical system consisted of a Waters LC (model 2487, Waters Corporation, Milford) equipped with two pumps, auto-sampler and a UV-detector. Waters Empower software was used for instrument access and data analysis. A mobile phase consisting of acetonitrile and water (55:45 v/v) was pumped through an Agilent column (C18, particle size 5 μM, 150 mm x 4.6 mm) at a flow rate of 1 ml/min, and 17-βE elution was monitored at 224 nm. The nanoemulsions were stored at 4^o^C after preparation. For stability, we evaluated the uniformity (appearance), size and zeta potential of the nanoemulsion droplets after 2 weeks, 4 weeks and 3 months of storage.


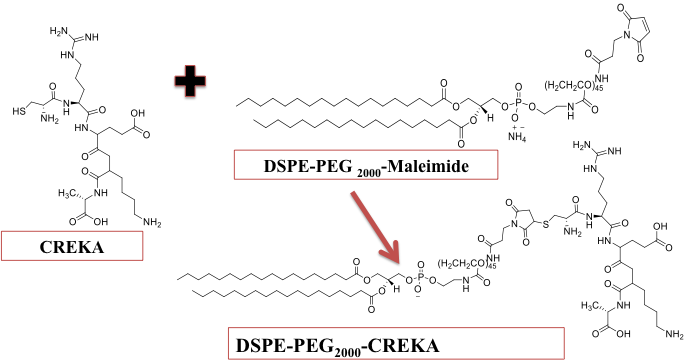
**Supplementary Figures**

**Figure A.** Synthesis of DSPE-PEG_2000_-CREKA Conjugate


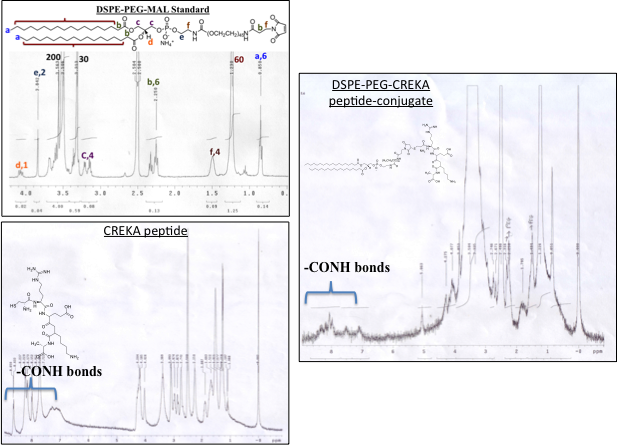


**Figure B.** NMR Scans of: (A) DSPE-PEG_2000_-maleimide; (B) CREKA peptide; and (C)DSPE PEG_2000_-CREKA conjugate

**Supplementary References**

1**.** Deshpande D, Janero DR, Amiji M (2013) Engineering of an omega-3 polyunsaturated fatty acid-containing nanoemulsion system for combination c6-ceramide and 17beta-estradiol delivery and bioactivity in human vascular endothelial and smooth muscle cells. Nanomedicine 9: 885-894

2. Sze A, Erickson D, Ren L, Li D (2003) Zeta-potential measurement using the smoluchowski equation and the slope of the current-time relationship in electroosmotic flow. J Colloid Interface Sci 261: 402-410
