# Supplementary material for: Therapeutic Efficacy of an ω-3-Fatty Acid-Containing 17-β Estradiol Nano-Delivery System against Experimental Atherosclerosis
Source: PLoS One. 2016 Feb 3;11(2):e0147337. doi: 10.1371/journal.pone.0147337 (PMC4740455; doi:10.1371/journal.pone.0147337)
Supplement: S2 File — Table A in S2 File reports comparison between no treatment, blank aqueous treatment and 17-βE solution treatment groups; Table B in S2 File reports comparison between no treatment, blank CREKA-peptide modified nanoemulsion and 17-βE loaded CREKA-peptide modified nanoemulsion treatment groups; and Table C in S2 File reports comparison between blank CREKA-peptide modified nanoemulsion, 17-βE loaded CREKA-peptide modified nanoemulsion and 17-βE loaded solution treatment groups respectively. (DOCX) [file pone.0147337.s004.docx]

**S2 File**. **Statistical comparison (ANOVA) of nitrate/nitrite levels measured across different treatment groups.**

**Table A**

| **COMPARISON** | | **Significance level** |
| --- | --- | --- |
| **TREATMENT A** | **TREATMENT B** |  |
| Media | Blank-aqueous | n.s. |
| Media | 17-βE Sol 0.001 μM | n.s. |
| Media | 17-βE Sol 0.01 μM | p < 0.01 |
| Media | 17-βE Sol 0.1 μM | p < 0.01 |
| Media | 17-βE Sol 0.3 μM | p < 0.001 |
| Media | 17-βE Sol 1 μM | p < 0.001 |
| Blank-aqueous | 17-βE Sol 0.001 μM | n.s. |
| Blank-aqueous | 17-βE Sol 0.01 μM | n.s. |
| Blank-aqueous | 17-βE Sol 0.1 μM | p < 0.01 |
| Blank-aqueous | 17-βE Sol 0.3 μM | p < 0.01 |
| Blank-aqueous | 17-βE Sol 1 μM | n.s. |

**Table A.** Statistical comparison (ANOVA) of nitrate/nitrite levels measured between no treatment, blank aqueous treatment and 17-βE solution treatment groups. GraphPad Prism^®^ software was used to compare treatment A (column 1) to treatment B (column 2) and significance level has been reported in the table

**Table B**

| **COMPARISON** | | **Significance level** |
| --- | --- | --- |
| **TREATMENT A** | **TREATMENT B** |  |
| Media | 17-βE NE 0.001 μM | p < 0.05 |
| Media | 17-βE NE 0.01 μM | p < 0.05 |
| Media | 17-βE NE 0.1 μM | p < 0.01 |
| Media | 17-βE NE 0.3 μM | p < 0.001 |
| Media | 17-βE NE 1 μM | p < 0.001 |
| Media | Blank NE 0.001 μM | n.s. |
| Media | Blank NE 0.01 μM | p < 0.01 |
| Media | Blank NE 0.1 μM | n.s. |
| Media | Blank NE 0.3 μM | n.s. |
| Media | Blank NE 1 μM | p < 0.001 |

**Table B**. Statistical comparison (ANOVA) of nitrate/nitrite levels measured between no treatment, blank CREKA-peptide modified nanoemulsion and 17-βE loaded CREKA-peptide modified nanoemulsion treatment groups respectively. GraphPad Prism^®^ software was used to compare treatment A (column 1) to treatment B (column 2) and significance level has been reported in the table

| **COMPARISON** | | **Significance level** |
| --- | --- | --- |
| **TREATMENT A** | **TREATMENT B** |  |
| Blank NE 0.001 μM | 17-βE NE 0.001 μM | n.s. |
| Blank NE 0.01 μM | 17-βE NE 0.01 μM | n.s. |
| Blank NE 0.1 μM | 17-βE NE 0.1 μM | p < 0.05 |
| Blank NE 0.3 μM | 17-βE NE 0.3 μM | p < 0.001 |
| Blank NE 1 μM | 17-βE NE 1 μM | n.s. |
| 17-βE NE 0.001 μM | 17-βE Sol 0.001 μM | n.s. |
| 17-βE NE 0.01 μM | 17-βE Sol 0.01 μM | n.s. |
| 17-βE NE 0.1 μM | 17-βE Sol 0.1 μM | n.s. |
| 17-βE NE 0.3 μM | 17-βE Sol 0.3 μM | n.s. |
| 17-βE NE 1 μM | 17-βE Sol 1 μM | n.s. |

**Table C**

**Table C**. Statistical comparison (ANOVA) of nitrate/nitrite levels measured between blank CREKA-peptide modified nanoemulsion, 17-βE loaded CREKA-peptide modified nanoemulsion and 17-βE loaded solution treatment groups respectively. GraphPad Prism^®^ software was used to compare treatment A (column 1) to treatment B (column 2) and significance level has been reported in the table
