# Supplementary material for: Therapeutic Efficacy of an ω-3-Fatty Acid-Containing 17-β Estradiol Nano-Delivery System against Experimental Atherosclerosis
Source: PLoS One. 2016 Feb 3;11(2):e0147337. doi: 10.1371/journal.pone.0147337 (PMC4740455; doi:10.1371/journal.pone.0147337)
Supplement: S3 File — Table A in S3 File reports comparison of lesion area measured across the different study groups; Table B in S3 File reports plaque lipid area measured across the different study groups; Table C in S3 File reports plaque SMC-actin stained area measured across the different study groups and Table D in S3 File reports plaque elastin stained area measured across the different study groups respectively. (DOCX) [file pone.0147337.s005.docx]

**S5 File. Statistical comparison (ANOVA) of lesions area and lesion content analysis:** Table A in S3 file reports comparison of lesion area measured across the different study groups; Table B in S3 file reports plaque lipid area measured across the different study groups; Table C in S3 file reports plaque SMC-actin stained area measured across the different study groups and Table D in S3 file reports plaque elastin stained area measured across the different study groups respectively.

**Table A**

| **COMPARISON** | | **Significance level** |
| --- | --- | --- |
| **TREATMENT A** | **TREATMENT B** |  |
| Control (Untreated) | 17-βE solution | p < 0.05 |
| Control (Untreated) | 17-βE NE | p < 0.05 |
| Control (Untreated) | Blank NE | n.s. |
| 17-βE solution | 17-βE NE | n.s. |
| 17-βE solution | Blank NE | n.s. |
| 17-βE NE | Blank NE | n.s. |

| **COMPARISON** | | **Significance level** |
| --- | --- | --- |
| **TREATMENT A** | **TREATMENT B** |  |
| Control (Untreated) | 17-βE solution | n.s. |
| Control (Untreated) | 17-βE NE | p < 0.05 |
| Control (Untreated) | Blank NE | n.s. |
| 17-βE solution | 17-βE NE | n.s. |
| 17-βE solution | Blank NE | n.s. |
| 17-βE NE | Blank NE | n.s. |

**Table A.** Statistical comparison (ANOVA) of lesion area measured between no treatment, 17-βE solution, blank CREKA-peptide modified nanoemulsion and 17-βE loaded CREKA-peptide modified nanoemulsion treatment groups respectively. GraphPad Prism^®^ software was used to compare treatment A (column 1) to treatment B (column 2) and significance level has been reported in the table.

**Table B**

**Table B.** Statistical comparison (ANOVA) of plaque lipid area measured between no treatment, 17-βE solution, blank CREKA-peptide modified nanoemulsion and 17-βE loaded CREKA-peptide modified nanoemulsion treatment groups respectively. GraphPad Prism^®^ software was used to compare treatment A (column 1) to treatment B (column 2) and significance level has been reported in the table

**Table C**

| **COMPARISON** | | **Significance level** |
| --- | --- | --- |
| **TREATMENT A** | **TREATMENT B** |  |
| Control (Untreated) | 17-βE solution | n.s. |
| Control (Untreated) | 17-βE NE | n.s. |
| Control (Untreated) | Blank NE | n.s. |
| 17-βE solution | 17-βE NE | n.s. |
| 17-βE solution | Blank NE | n.s. |
| 17-βE NE | Blank NE | n.s. |

**Table C.** Statistical comparison (ANOVA) of plaque SMC-actin stained area measured between no treatment, 17-βE solution, blank CREKA-peptide modified nanoemulsion and 17-βE loaded CREKA-peptide modified nanoemulsion treatment groups respectively. GraphPad Prism^®^ software was used to compare treatment A (column 1) to treatment B (column 2) and significance level has been reported in the table

**Table D**

| **COMPARISON** | | **Significance level** |
| --- | --- | --- |
| **TREATMENT A** | **TREATMENT B** |  |
| Control (Untreated) | 17-βE solution | n.s. |
| Control (Untreated) | 17-βE NE | n.s. |
| Control (Untreated) | Blank NE | n.s. |
| 17-βE solution | 17-βE NE | n.s. |
| 17-βE solution | Blank NE | n.s. |
| 17-βE NE | Blank NE | n.s. |

**Table D.** Statistical comparison (ANOVA) of plaque elastin stained area measured between no treatment, 17-βE solution, blank CREKA-peptide modified nanoemulsion and 17-βE loaded CREKA-peptide modified nanoemulsion treatment groups respectively. GraphPad Prism^®^ software was used to compare treatment A (column 1) to treatment B (column 2) and significance level has been reported in the table
