# Supplementary material for: Therapeutic Efficacy of an ω-3-Fatty Acid-Containing 17-β Estradiol Nano-Delivery System against Experimental Atherosclerosis
Source: PLoS One. 2016 Feb 3;11(2):e0147337. doi: 10.1371/journal.pone.0147337 (PMC4740455; doi:10.1371/journal.pone.0147337)
Supplement: S4 File — Table A in S4 File represents fold change values and statistical analysis of gene expression in apoE-/- mice receiving high fat diet and the 17-βE solution, 17-βE nanoemulsion and blank nanoemulsion treatment groups measured relative to gene expression in wild type mice. Table B in S4 File represents statistical analysis of total plasma triglycerides measured across the different study groups and Table C in S4 File represents statistical analysis of total plasma cholesterol measured across the different study groups. (DOCX) [file pone.0147337.s006.docx]

**S4 File: Detailed statistical analysis of the effect of the 17-βE-CREKA nanoemulsion on the expression of atherosclerosis-related genes within aorta and circulating plasma lipids.** Table A in S4 file represents fold change values and statistical analysis of gene expression in apoE^-/-^ mice receiving high fat diet and the 17-βE solution, 17-βE nanoemulsion and blank nanoemulsion treatment groups measured relative to gene expression in wild type mice. Table B in S4 file represents statistical analysis of total plasma triglycerides measured across the different study groups and Table C in S4 file represents statistical analysis of total plasma cholesterol measured across the different study groups.

**Table A:**

|  | **Untreated** | | **17-βE Solution** | | **17-βE Nanoemulsion** | | **Blank Nanoemulsion** | |
| --- | --- | --- | --- | --- | --- | --- | --- | --- |
|  | **Fold Change** | **P value** | **Fold Change** | **P value** | **Fold Change** | **P value** | **Fold Change** | **P value** |
| Ccl2 | 4.93 | < 0.001 | 2.61 | n.s. | 1.95 | n.s. | 3.53 | < 0.05 |
| ICAM-1 | 2.57 | < 0.001 | 0.43 | < 0.05 | 0.43 | < 0.05 | 0.46 | n.s. |
| Ifng | 9.31 | < 0.001 | 1.59 | n.s. | 0.54 | n.s. | 0.86 | n.s. |
| Itgax | 9.02 | < 0.001 | 0.71 | n.s. | 0.77 | n.s. | 1.21 | n.s. |
| Msr1 | 2.61 | < 0.001 | 1.51 | n.s. | 0.73 | n.s. | 0.95 | n.s. |
| Selplg | 5.33 | < 0.001 | 0.95 | n.s. | 0.62 | n.s. | 0.80 | n.s. |
| Tnf | 6.40 | < 0.001 | 2.11 | < 0.05 | 0.66 | n.s. | 0.84 | n.s. |
| VCAM-1 | 7.40 | < 0.001 | 1.63 | n.s. | 0.86 | n.s. | 0.90 | n.s. |
| IL6 | 4.22 | < 0.001 | 1.36 | n.s. | 0.50 | < 0.05 | 1.25 | n.s. |

**Table A.** Fold change values of gene expression in apoE^-/-^ mice receiving high fat diet and the 17-βE solution, 17-βE nanoemulsion and blank nanoemulsion treatment groups measured relative to gene expression in wild type mice. Data analysis was performed using the RT^2^  Profiler PCR Array Data Analysis system provided by SABioscinces (Valencia, CA)

**Table B**

**Table B**. Statistical comparison (ANOVA) of the total plasma triglyceride measured in the untreated, 17-βE solution, 17-βE nanoemulsion and blank nanoemulsion treatment groups. GraphPad Prism^®^ software was used for the comparison and the statistical significance has been reported in the table.

| **COMPARISON (Final Week)** | | **Significance level** |
| --- | --- | --- |
| **TREATMENT A** | **TREATMENT B** |  |
| Control (Untreated) | 17-βE Solution | p < 0.05 |
| Control (Untreated) | 17-βE NE | n.s. |
| Control (Untreated) | Blank NE | n.s. |
| 17-βE Solution | 17-βE NE | n.s. |
| 17-βE Solution | Blank NE | p < 0.05 |
| Blank NE | 17-βE NE | n.s. |

**Table C**

| **COMPARISON (Final Week)** | | **Significance level** |
| --- | --- | --- |
| **TREATMENT A** | **TREATMENT B** |  |
| Control (Untreated) | 17-βE Solution | n.s. |
| Control (Untreated) | 17-βE NE | p < 0.05 |
| Control (Untreated) | Blank NE | n.s. |
| 17-βE Solution | 17-βE NE | n.s. |
| 17-βE Solution | Blank NE | p < 0.05 |
| Blank NE | 17-βE NE | p < 0.01 |

**Table C**. Statistical comparison (ANOVA) of the total plasma cholesterol measured in the untreated, 17-βE solution, 17-βE nanoemulsion and blank nanoemulsion treatment groups. GraphPad Prism^®^ software was used for the comparison and the statistical significance has been reported in the table.
